# Multiferroic Compounds with Double-Perovskite Structures

**DOI:** 10.3390/ma4010153

**Published:** 2011-01-07

**Authors:** Yuichi Shimakawa, Masaki Azuma, Noriya Ichikawa

**Affiliations:** Institute for Chemical Research, Kyoto University, Uji, Kyoto 611-0011, Japan; E-Mails: azuma.m.ab@m.titech.ac.jp (M.A.); nori@scl.kyoto-u.ac.jp (N.I.)

**Keywords:** double-perovskite structure, ferromagnetic and ferroelectric properties, high-pressure synthesized bulk, epitaxially grown thin film, artificial superlattice

## Abstract

New multiferroic compounds with double-perovskite structures were synthesized. Bi_2_NiMnO_6_ was synthesized in bulk form by high-pressure synthesis and also in a thin-film form by epitaxial growth. The material showed both ferromagnetic and ferroelectric properties, *i.e.*, the multiferroic property at low temperature. Bi_2_FeCrO_6_ was also fabricated in a (1 1 1) oriented BiFeO_3_/BiCrO_3_ artificial superlattice, with a 1/1 stacking period. The superlattice film showed ferromagnetic behavior and polarization switching at room temperature. In the compounds, Bi^3+^ ion, located at the A site in the perovskite structure, caused ferroelectric structural distortion, and the B-site ordering of the Ni^2+^ and Mn^4+^ ions (Fe^3+^ and Cr^3+^ ions) in a rock-salt configuration led to ferromagnetism according to the Kanamori-Goodenough rule.

## 1. Introduction

Multiferroic materials exhibit more than one ferroic order. In particular, materials, in which ferromagnetic and ferroelectric orders coexist, attract a lot of attention in fundamental physics and chemistry [1,2,3]. Potential technological applications, such as nonvolatile memories and sensors, also generate research of the ferromagnetic and ferroelectric materials [1,3].

Of the multiferroic oxides, BiFeO_3_ perovskite is drawing a lot of attention. Bulk BiFeO_3_ intrinsically shows multiferroic properties; *i.e.*, ferroelectric polarization and weak magnetism at room temperature [4]. The rhombohedrally distorted perovskite structure leads to spontaneous polarization below the ferroelectric transition temperature (*T*_C_ = 1,103 K). In an epitaxially-grown BiFeO_3_ thin film, large spontaneous polarization (~100 μC/cm^2^) was observed at room temperature [5,6]. The magnetic property originates from a residual moment of a spin canting of the antiferromagnetically ordered spin structure below the antiferromagnetic Nèel temperature (*T*_N_ = 643 K).

Recent discoveries of strong interplay between magnetism and ferroelectricity in TbMnO_3_ [7] and TbMn_2_O_5_ [8] have also stimulated a lot of interest in strong coupling between ferroelectric and ferromagnetic order parameters. In these compounds (non-)collinear spiral magnetic structures induce ferroelectric spontaneous polarization [9] and the significant changes in dielectric properties under applied magnetic fields were observed.

**Figure 1:**
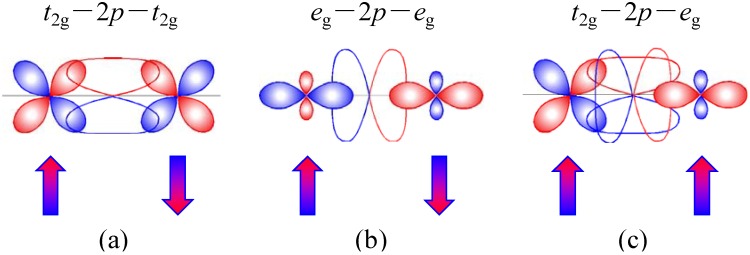
Magnetic interaction between octahedral site transition-metal ions via an oxygen ion according to the Kanamori-Goodenough rule (180° cation-anion-cation interactions). **(a)** Antiferromagnetic interaction of M(*t*_2g_)-O-M(*t*_2g_). Half filled *t*_2g_ orbitals of cations overlapping *p*π produce the antiferromagnetic interaction; **(b)** Antiferromagnetic interaction of M’(*e*_g_)-O-M’(*e*_g_). Half filled *e*_g_ orbitals overlapping *p*σ mediate the strong antiferromagnetic interaction; **(c)** Ferromagnetic interaction of M(*t*_2g_)-O-M’(*e*_g_).

In searching for new multiferroic compounds, we focused on Bi- or Pb-containing compounds with perovskite structures. Bi^3+^ and Pb^2+^ ions are often located at the A site in the ABO_3_ perovskite structure and 6s lone pair of electrons of the ions cause ferroelectric structural distortion. The strong covalent character of Bi/Pb-O bonds in the structure also stabilizes a noncentrosymmetric structure [10,11]. Indeed, some Bi- or Pb-containing compounds such as PbTiO_3_ show large spontaneous ferroelectric polarizations. BiCoO_3_ [12] and PbVO_3_ [13] are also tetragonal perovskites with large distortions (*c/a* > 1.2) and can be ferroelectric, though the polarization changes were not confirmed due to the large coercive fields. Concerning the magnetism, on the other hand, BiFeO_3_, BiCoO_3_, and PbVO_3_ are all intrinsically antiferromagnets. This is due to an antiferromagnetic superexchange interaction induced between transition-metal ions via oxygen ions according to the Kanamori-Goodenough rule. For example, half filled *t*_2g_ orbitals of cations directed towards the anion intermediary overlap *p*π and produce the antiferromagnetic interaction between the transition-metal ions as illustrated in Figure 1(a). Also, half filled *e*_g_ orbitals overlapping *p*σ mediate the strong antiferromagnetic interaction (Figure 1(b)). In order to give rise to an intrinsic strong ferromagnetic interaction, two transition-metal ions are needed; one with *e*_g_ electrons (M) and another without *e*_g_ electrons (M’), in an M-O-M’ linear arrangement (Figure 1(c)). Such a linear arrangement is achieved in a double perovskite structure, in which M and M’ are ordered in a rock-salt manner at the B site in the perovskite structure. Indeed, La_2_NiMnO_6_ [14,15,16], La_2_CoMnO_6_, [14,15,17], and La_2_CuMnO_6_ [14,18] with the double-perovskite structures show ferromagnetic behaviors. We thus focused on (Bi/Pb)_2_MM’O_6_ double-perovskite compounds as possible ferroelectric and ferromagnetic materials.

We succeeded in synthesizing a few new multiferroic materials which show both ferromagnetic and ferroelectric orders. In this article, those examples are reviewed. The first result concerns Bi_2_NiMnO_6_ [19,20,21]. The crystal structure of the compound is the double perovskite where Bi^3+^ ions are located at the A site and magnetic Ni^2+^ and Mn^4+^ ions are ordered at the B site. The compound was obtained in bulk form by high-pressure synthesis and also in a thin-film form by epitaxial growth. Both ferroelectric and ferromagnetic properties were observed. An attempt to make another multiferroic material, Bi_2_CrFeO_6_, is also highlighted [22]. Although ordered materials with Cr^3+^ and Fe^3+^ are difficult to synthesize, Bi_2_CrFeO_6_ prepared in an artificial superlattice is a promising compound.

## 2. Results and Discussion

### 2.1. Bi_2_NiMnO_6_ High-Pressure Synthesized Bulk

**Figure 2:**
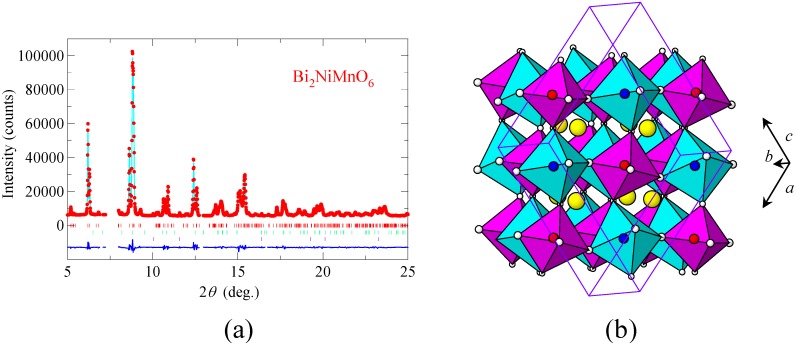
**(a)** Synchrotron X-ray powder diffraction pattern of Bi_2_NiMnO_6_ and the Rietveld refinement profile. The observed (red dot), calculated (light-blue line), and difference (bottom blue line) patterns are shown. Bragg reflections are indicated by tick marks. Diffractions from impurities (0.65 *wt*% Bi_2_(CO_3_)O_2_ and 1.92 *wt*% NiO) are also included in the refinement; **(b)** Crystal structure of Bi_2_NiMnO_6_. The blue octahedra correspond to NiO_6_ and the red octahedra correspond to MnO_6_. Bi ions are shown in yellow spheres. The monoclinic unit cell is superimposed.

Bi_2_NiMnO_6_ cannot be synthesized under an ambient-pressure condition. However, we found that the compound is obtained by means of high-pressure synthesis. Although small amounts of impurities were included, nearly single phase sample (>97%) was obtained by the synthesis at 6 GPa and 800 °C [19]. Figure 2(a) shows the synchrotron X-ray powder diffraction pattern taken at room temperature and the result of the structure refinement with the Rietveld method. The diffraction pattern shows that the material is crystallized with the Bi_2_NiMnO_6_ double-perovskite structure, with the monoclinic (space group *C*2) unit cell of *a* = 9.4646(4) Å, *b* = 5.4230(2) Å, *c* = 9.5431(4) Å, and *β* = 107.823(2)°. At the initial stage of the refinement, Ni^2+^ and Mn^4+^ were randomly located at the three crystallographic sites (M(1), M(2), and M(3), with multiplicities of 2, 4, and 2, respectively) in the monoclinic structure. It was found that the M-O bond lengths were considerably shorter for the M(2) site than M(1) and M(3), so the small Mn^4+^ ion was assigned to the M(2) site, and large Ni^2+^ ions were assigned to M(1) and M(3) at the final stage. The refined structural parameters are summarized in Table 1. The results of the structural analysis revealed that Bi^3+^ is located at the A site and that Ni^2+^ and Mn^4+^ ions are ordered in a rock-salt configuration at the B site as shown in Figure 2(b). Bond valence sums [23] calculated from the refined structural parameters were 2.14 and 2.17 for the Ni sites, and 3.62 for the Mn site, which also support the structure model. The NiO_6_ and MnO_6_ octahedra of Bi_2_NiMnO_6_ are rather isotropic, reflecting the absence of Jahn-Teller distortions, which is also consistent with the Ni^2+^ (*t*_2g_^6^*e*_g_^2^) and Mn^4+^ (*t*_2g_^3^) oxidation states of the transition-metal ions in the compound.

**Table 1:** Refined structure parameters of Bi_2_NiMnO_6_ at room temperature. Space group *C*2, *a* = 9.4646(4) Å, *b* = 5.4230(2) Å, *c* = 9.5431(4) Å, and *β* = 107.823(2) °. *R*_wp_ = 4.79% and *R*_I_ = 0.64%. The same thermal parameter values (*B*) were given for Bi(1) and Bi(2), and also for Ni and Mn, respectively. The *B* value of O was fixed during the refinement.

atom	site	*x*	*y*	*z*	*B* (Å^2^)
Bi(1)	4*c*	0.133(1)	−0.023(12)	0.378(1)	0.672(5)
Bi(2)	4*c*	0.369(1)	0.035(12)	0.123(1)	0.672 (=Bi(1))
Ni(1)	2*a*	0	0	0	0.40(7)
Ni(2)	2*b*	0.5	0.015(2)	0.5	0.40 (=Ni(1))
Mn	4c	0.243(3)	0.013(13)	0.749(3)	0.40 (=Ni(1))
O(1)	4*c*	0.111(5)	−0.061(15)	0.849(6)	0.8
O(2)	4*c*	0.420(4)	0.042(14)	0.680(5)	0.8
O(3)	4*c*	0.146(9)	0.276(18)	0.636(9)	0.8
O(4)	4*c*	0.333(4)	0.242(14)	0.413(5)	0.8
O(5)	4*c*	0.377(5)	0.204(12)	0.899(5)	0.8
O(6)	4*c*	0.162(8)	0.216(17)	0.126(9)	0.8

**Figure 3:**
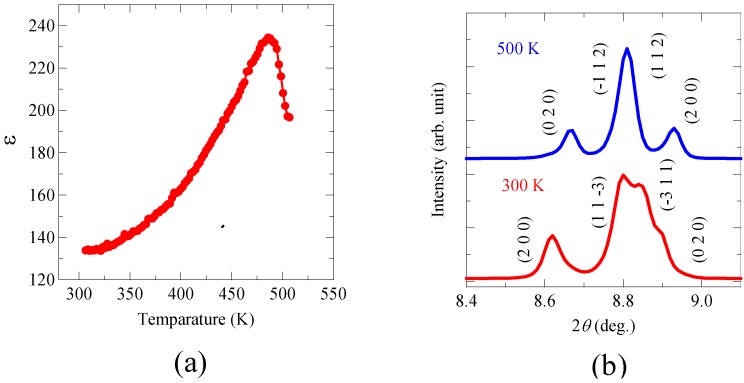
**(a)** Temperature dependence of dielectric constant of Bi_2_NiMnO_6_ measured at 10 kHz. A peak at 485 K indicates the ferroelectric Curie temperature; **(b)** Powder X-ray diffraction patterns taken at 300 and 500 K. The indices of the diffraction pattern at 300 K are for the noncentrosymmetric *C*2 structure, while those at 500 K for the centrosymmetric monoclinic *P*2_1_/*n* structure. The results clearly show the ferroelectric transition at 485 K of Bi_2_NiMnO_6_.

The noncentrosymmetric *C*2 space group of the compound allows a spontaneous polarization along the *b* axis, and a calculation based on an ionic point charge model with the obtained structural parameters gives a polarization of 20 μC/cm^2^. The Bi^3+^ ion at the A site in the perovskite structure should give rise to significant anisotropic structural distortion in the material. The temperature dependence of the dielectric constant, ε, shown in Figure 3(a) also exhibits a typical ferroelectric behavior with a ferroelectric Curie temperature of about 485 K. Correspondingly, the crystal structure changes from the room-temperature noncentrosymmetric phase to a high-temperature centrosymmetric phase with a monoclinic structure (space group, *P*2_1_/*n; a* = 5.4041(2) Å, *b* = 5.5669(1) Å, *c* = 7.7338(2) Å, and *β* = 90.184(2) °) above the Curie temperature as shown in Figure 3(b). Thus, the structural and dielectric measurement results confirm that Bi_2_NiMnO_6_ is ferroelectric.

**Figure 4:**
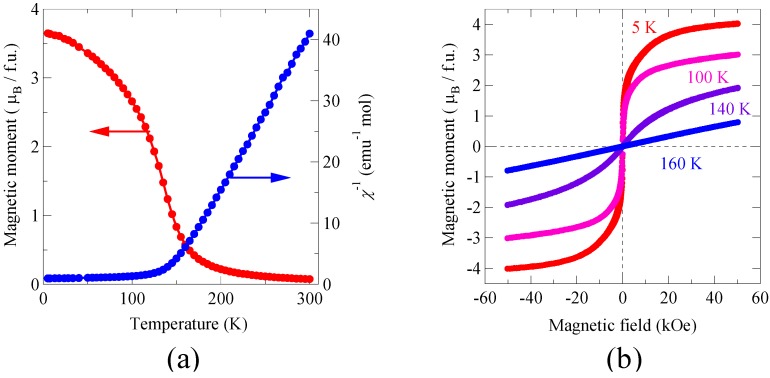
**(a)** Temperature dependence of the magnetization and inverse magnetic susceptibility of Bi_2_NiMnO_6_ measured under an external field of 10 kOe. The increase in the magnetic moment below 140 K suggests the ferromagnetic transition at that temperature; **(b)** Magnetization curves measured from 5 to 160 K. The ferromagnetic hysteresis was observed for the measurements at 5, 100, and 140 K.

The rock-salt configuration of the Ni^2+^ and Mn^4+^ ions in the double-perovskite structure gives the magnetic exchange path of Ni^2+^-O-Mn^4+^. Since a Ni^2+^ ion has the *t*_2g_^6^*e*_g_^2^ electron configuration with *e*_g_ electrons while a Mn^4+^ ion has no *e*_g_ electron (*t*_2g_^3^), a ferromagnetic interaction is expected between the adjacent spins according to the Kanamori-Goodenough rule. Figure 4(a) shows the temperature dependence of the magnetic moment and inverse magnetic susceptibility measured under an external field of 10 kOe. Magnetization curves measured at temperatures from 5 to 160 K are also shown in Figure 4(b). The material shows a typical ferromagnetic behavior below 140 K. The magnetic susceptibility above the transition temperature obeys the Curie-Weiss law and the Weiss constant is about 140 K, which also suggests the ferromagnetic interaction between the Ni^2+^ and Mn^4+^ spins. The observed saturated magnetization at 5 K is 4.1 μ_B_/f.u., which is close to 5 μ_B_/f.u. expected from the ferromagnetic ordering of Ni^2+^ (*S* = 1) and Mn^4+^ (*S* = 3/2). This is in sharp contrast with the BiFeO_3_ compound, in which magnetic Fe^3+^ spins are aligned antiferromagnetically through the superexchange interaction. The slight deviation from the expected saturated moment is probably due to small amount of antisite disorder of the Ni^2+^ and Mn^4+^ ions. The resulting Ni-O-Ni and Mn-O-Mn magnetic paths produce antiferromagnetic interactions and thus reduce the saturated magnetization. It should be noted that a sample obtained by quenching from 800 °C after the high-pressure synthesis resulted in the random mixing of Ni and Mn, and that a substantial decrease of the saturated magnetic moment was observed. The magnetic measurement results clearly show that Bi_2_NiMnO_6_ is ferromagnetic. Therefore, we can conclude that the material is a multiferroic, and both ferroelectric and ferromagnetic properties are observed below 140 K.

In Bi_2_NiMnO_6_ the ferroelectric transition temperature is 485 K and the ferromagnetic transition temperature is 140 K, and the two ferroic orderings occur independently. Even though, both ferroelectric and ferromagnetic orderings, *i.e.*, the multiferroic property, appear below 140 K.

### 2.2. Bi_2_NiMnO_6_ Epitaxially Grown Thin Film

As described above, Bi_2_NiMnO_6_ is a meta-stable phase synthesized under high-pressure conditions. The compound can also be obtained in a thin-film form by using epitaxial strain from the substrate lattice. A Bi_2_NiMnO_6_ thin film was thus grown epitaxially on a SrTiO_3_ substrate by a pulsed laser deposition method [20].

**Figure 5:**
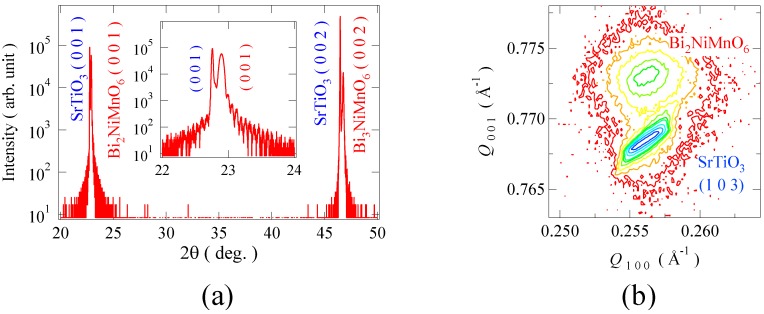
**(a)** X-ray diffraction patterns of Bi_2_NiMnO_6_ thin film grown on SrTiO_3_ substrate; **(b)** Logarithmic intensity reciprocal space map around (1 0 3) reflection of the thin film. In addition to the SrTiO_3_ substrate peaks, Bragg reflections of the perovskite Bi_2_NiMnO_6_ thin film are clearly seen and the results also confirm the epitaxial growth of the film on the substrate lattice.

Figure 5(a) shows the *θ*-2*θ* X-ray diffraction pattern of the Bi_2_NiMnO_6_ thin film grown on the SrTiO_3_ (0 0 1) substrate. In the diffraction pattern, only (0 0 *l*) reflections of the perovskite structure are observed, and the out-of-plane lattice parameter, *c*, is ascertained to be 3.87 Å from the data. Figure 5(b) shows a logarithmic intensity map in reciprocal lattice space around the (1 0 3) reflection for the Bi_2_NiMnO_6_ thin film. We can see that the in-plane lattice parameter of the film matches that of the substrate perovskite structure (*a* = 3.91 Å) while the out-of-plane lattice parameter (3.87 Å) is smaller than the substrate cell. The results of the X-ray diffraction measurements indicate that the strained pseudo-tetragonal Bi_2_NiMnO_6_ thin film epitaxially grows on the SrTiO_3_ substrate. In addition, the reflection high energy diffraction (RHEED) pattern strongly suggests a double-perovskite structure. Electron beam incidence from the [1 1 0] direction gives weak streaks of the 2-fold superstructure, whereas the [1 0 0] incidence shows no such a superstructure reflection. These results imply that the synthesized Bi_2_NiMnO_6_ thin film has a √2*a*_p_×√2*a*_p_ superstructure, which originates from a rock-salt-type ordering of the B site ions in the double-perovskite structure.

**Figure 6:**
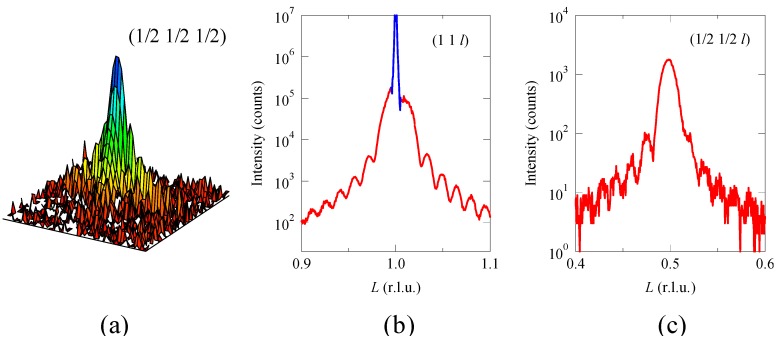
**(a)** Synchrotron X-ray logarithmic intensity reciprocal space map around (1/2 1/2 1/2) reflection of Bi_2_NiMnO_6_ thin film grown on SrTiO_3_ substrate; **(b)** (1 1 1) and **(c)** (1/2 1/2 1/2) intensity profiles along the *L* direction of the thin film. A sharp diffraction peak (blue) in (b) originates from the SrTiO_3_ substrate. A weak intensity of the (1/2 1/2 1/2) superstructure reflection, which is only 1% of the (1 1 1) fundamental reflection, can be observed in the synchrotron X-ray diffraction, confirming the rock-salt type ordering of the Ni^2+^ and Mn^4+^ ions in the Bi_2_NiMnO_6_ double perovskite structure.

A clear evidence of the rock-salt configuration of the Ni^2+^ and Mn^4+^ ions in the perovskite structure is also seen in the (1/2 1/2 1/2) superstructure reflection in a reciprocal lattice space intensity map obtained by synchrotron X-ray diffraction as shown in Figure 6. Here, the reciprocal-space coordinates correspond to the cubic SrTiO_3_ substrate. The observed superstructure reflection is consistent with that from a 2*a* × 2*a* × 2*c* ordered structure. It is noted that the observed intensity of the superstructure reflection is about 1×10^3^ cps, which is only 1% of the (1 1 1) fundamental reflection intensity. And the weak intensity of the (1/2 1/2 1/2) superstructure reflection is well reproduced from a simple ordered structure model where Ni^2+^ and Mn^4+^ ions are ordered in the rock-salt configuration in the perovskite structure. The calculated intensities of the (1/2 1/2 1/2) and (1 1 1) reflections for the rock-salt arrangement of the Ni and Mn ions are 94 and 33,055, respectively, for the maximum peak intensity of 100,000 for the (1 0 1) reflection. Note that the calculated (1/2 1/2 1/2) diffraction intensity for the random arrangement of the Ni and Mn ions at the B site in the perovskite structure is zero. Also note that the Laue’s-intensity oscillation is observed on the superstructure diffraction peak with the same interval as that observed on the (1 1 1) reflection, confirming that both (1 1 1) and (1/2 1/2 1/2) diffraction peaks originate from the structure of the grown Bi_2_NiMnO_6_ film.

**Figure 7:**
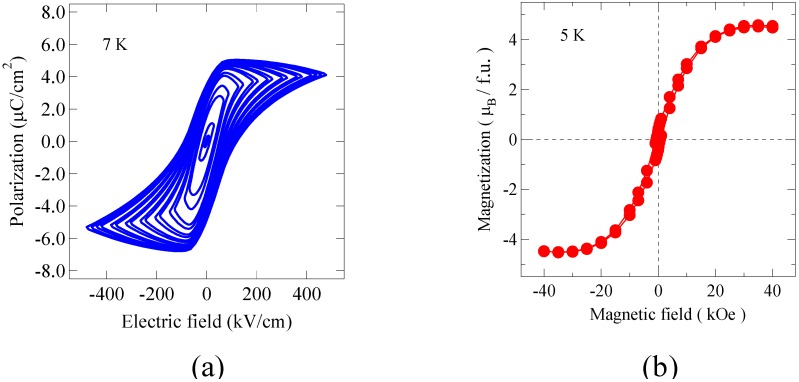
**(a)** Ferroelectric hysteresis curves of the Bi_2_NiMnO_6_ thin film measured at 7 K of the film. Clear polarization hysteresis loops confirm the ferroelectricity of the film; **(b)** Magnetization curve measured at 5 K of the thin film. A typical ferromagnetic behavior was seen.

The ferroelectric behavior of the Bi_2_NiMnO_6_ thin film is confirmed by the polarization hysteresis measured at 7 K as shown in Figure 7(a). Although the observed slightly distorted hysteresis loop includes an effect of leakage current, we can see the saturated polarization of about 5 μC/cm^2^ above 80 kV/cm. The observed polarization is quite small compared to the polarization of about 20 μC/cm^2^ calculated from the refined bulk crystal structure. If the essential crystal structure of the thin film is similar to that of bulk with the *C2* noncentrosymmetric space group, the polarization vector should be along the [1 1 1] direction of the simple perovskite structure, and thus the observed ferroelectric polarization of the film should be a projection along the out-of-plane direction. This may explain the difference between the bulk and thin film in the polarization value. Figure 7(b) shows a magnetization behavior of the Bi_2_NiMnO_6_ film at 5 K. The observed saturated magnetization is 4.2 μ_B_/f.u., which is close to the 5 μ_B_/f.u. that would be expected from the ferromagnetic ordering of Ni^2+^ (*S* = 1) and Mn^4+^ (*S* = 3/2) spins. The magnetization is also consistent with the observed moment of the bulk sample synthesized under high pressure. The important point is that our Bi_2_NiMnO_6_ thin film shows both ferromagnetic and ferroelectric properties, that is the multiferroic property, at low temperatures.

**Figure 8:**
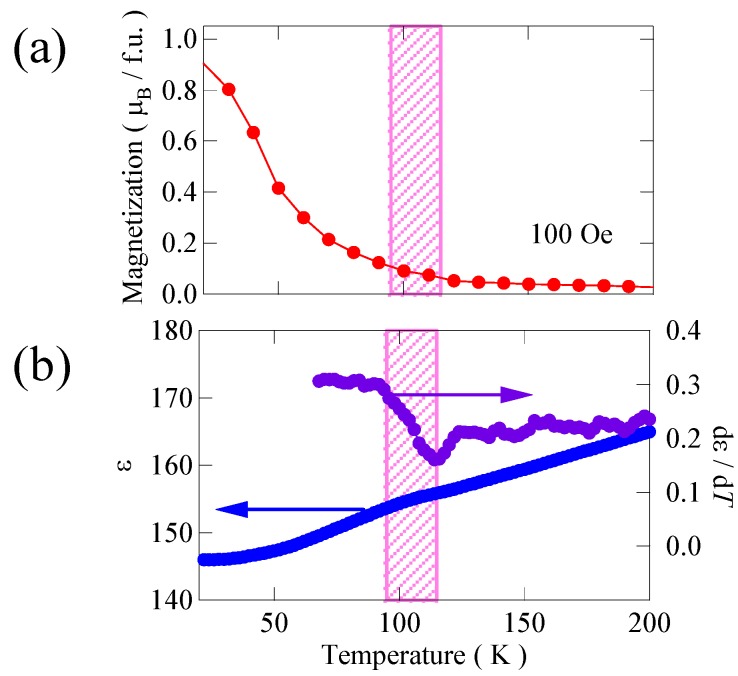
**(a)** Temperature dependence of magnetization of Bi_2_NiMnO_6_ thin film grown on SrTiO_3_ substrate; **(b)** Temperature dependence of dielectric constant and the temperature derivative of the film. An anomaly in ε near the ferromagnetic transition temperature (near 100 K indicated in pink) suggests some interplay between the ferromagnetic and ferroelectric interactions.

Then, we looked at the coupling between the ferroelectric and ferromagnetic properties of our Bi_2_NiMnO_6_ film by measuring the dielectric property around the ferromagnetic transition temperature. Figure 8(a) shows the temperature dependence of the magnetization of the Bi_2_NiMnO_6_ thin film measured under a 100 Oe magnetic field applied to the in-plane direction. The magnetization increases below about 100 K, suggesting that the ferromagnetic transition of the present Bi_2_NiMnO_6_ film is around 100 K. Although this transition temperature is lower than 140 K observed with the bulk, structural strain of the epitaxially grown film may change the ferromagnetic transition temperature. As shown in Figure 8(b), the observed dielectric constant shows a very small anomaly near the ferromagnetic transition temperature. Although the change is very small, the anomaly near the ferromagnetic transition temperature appears to show some interplay between the ferromagnetic and ferroelectric interactions. Thus, the coupling between ferromagnetic and ferroelectric interactions seems to exist in Bi_2_NiMnO_6_, but it is quite small.

### 2.3. Bi_2_FeCrO_6_ Artificial Superlattice Thin Film

**Figure 9:**
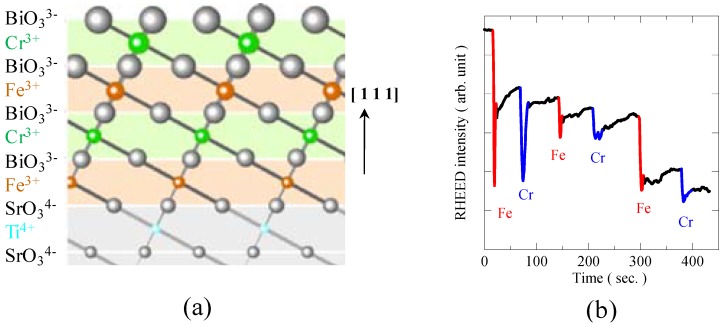
**(a)** Schematic figure of BiFeO_3_/BiCrO_3_ artificial superlattice grown on SrTiO_3_ (1 1 1) substrate. Monolayers of BiFeO_3_ and BiCrO_3_ are shown in orange and green, respectively. One-by-one stacking of the BiFeO_3_ and BiCrO_3_ monolayers along [1 1 1] direction produces the rock-salt configuration of Bi^3+^ and Cr^3+^ ions in the perovskite structure, so the Fe^3+^-O-Cr^3+^ magnetic path is expected to lead to the ferromagnetism; **(b)** RHEED intensity oscillation during the deposition of the 6-monolayer artificial superlattice thin film, confirming the two-dimensional layer-by-layer growth.

Another possible multiferroic compound is a double perovskite, Bi_2_FeCrO_6_, because electron configurations of Fe^3+^ and Cr^3+^ are *t*_2g_^3^*e*_g_^2^ and *t*_2g_^3^, respectively. However, a bulk sample synthesized under a high-pressure condition did not show large magnetization from the ferromagnetism [24]. The observed saturated magnetization of a thin film sample was also more than an order of magnitudes smaller than that expected from the ideal value of the ferromagnetic ordering of Fe^3+^ and Cr^3+^ spins [25]. These should result from disorder between Fe^3+^ and Cr^3+^ ions at the B site in the perovskite structure because they are isovalent and have similar ionic radii. We thus made a BiFeO_3_/BiCrO_3_ artificial superlattice with a 1/1 stacking period on a perovskite-structure SrTiO_3_ (1 1 1) substrate [22]. While BiFeO_3_ and BiCrO_3_ [26] are antiferromagnets, these 1/1 superlattice along [1 1 1] direction contain Fe-O-Cr bonds producing the rock-salt configuration in the perovskite structure (Figure 9(a)).

The 1/1 artificial superlattice of BiFeO_3_/BiCrO_3_ was grown epitaxially on a SrTiO_3_ substrate by a pulsed laser deposition method. Two-dimensional layer-by-layer growth was confirmed by monitoring the RHEED intensity oscillations *in situ* from the start of the deposition to the end. Figure 9(b) shows the change in RHEED intensity during the deposition of a 6-monolayer (ML) superlattice. So far, clear oscillations during the depositions for the growth of only 2 ML ([BiFeO_3_/BiCrO_3_]_1_), 4 ML ([BiFeO_3_/BiCrO_3_]_2_), and 6 ML ([BiFeO_3_/BiCrO_3_]_3_) were observed. Although for the sample identification it is difficult to obtain the X-ray diffraction patterns from such very thin film samples, the results of magnetic and ferroelectric property measurements appear to show multiferroic property of the prepared artificial superlattice.

**Figure 10:**
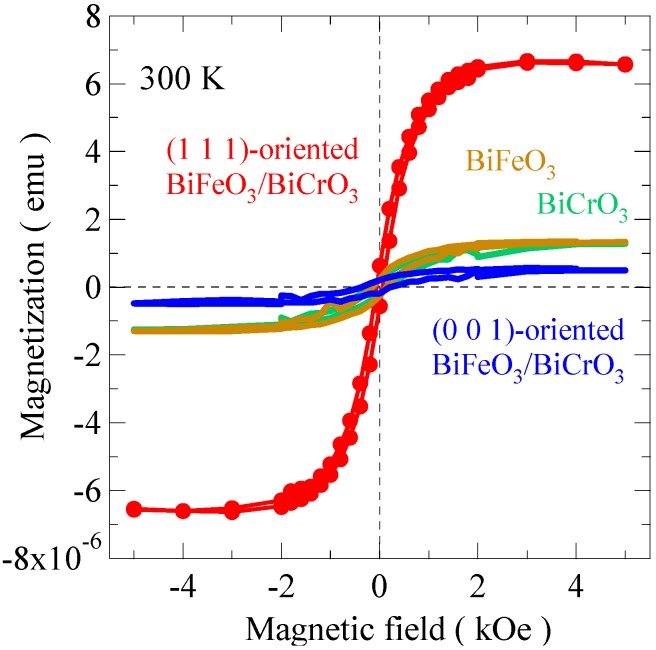
Magnetization curves measured at 300 K of 4-monolayer BiFeO_3_/BiCrO_3_ artificial superlattice grown on SrTiO_3_ (1 1 1) substrate (red). Those of 4-monolayer BiFeO_3_/BiCrO_3_ artificial superlattice grown on SrTiO_3_ (0 0 1) substrate (blue), and 4-monolayer BiFeO_3_ (brown) and 4-monolayer BiCrO_3_ (green) thin films grown on SrTiO_3_ (1 1 1) substrates are also shown. Only the (1 1 1)-oriented BiFeO_3_/BiCrO_3_ artificial superlattice shows the large magnetization, confirming the ferromagnetic property of the film.

Figure 10 shows the magnetization curves of the 4-ML ([BiFeO_3_/BiCrO_3_]_2_) artificial superlattice film measured at 300 K. Those of 4-ML BiFeO_3_ and 4-ML BiCrO_3_ grown on SrTiO_3_ (1 1 1), and that of a 4-ML ([BiFeO_3_/BiCrO_3_]_2_) artificial superlattice film grown on a SrTiO_3_ (0 0 1) substrate are also shown in the figure. The magnetization in the BiFeO_3_/BiCrO_3_ superlattices evidently depends on the orientations and the results are consistent with the Kanamori-Goodenough rule. The magnetization signals of the (0 0 1) superlattice are quite small and compatible with the extrinsic magnetization from the substrate. In the (0 0 1) superlattice the magnetic interaction between the monolayers is antiferromagnetic, so the stacking does not lead to ferromagnetism. The magnetic response from each single material film with the (1 1 1) orientation was also small. Therefore, the magnetization results strongly suggest that the BiFeO_3_/BiCrO_3_ artificial superlattice with the 1/1 stacking period along the [1 1 1] direction produced the ferromagnetic moment.

**Figure 11:**
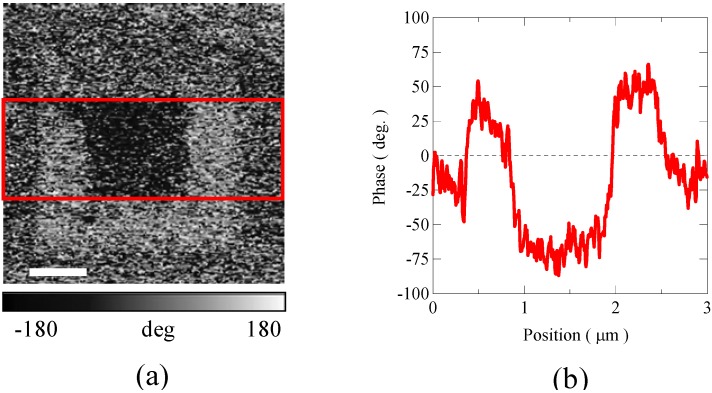
**(a)** Non-linear dielectric constant microscope phase image taken at room temperature of 6-monolayer BiFeO_3_/BiCrO_3_ artificial superlattice grown on SrTiO_3_ (1 1 1) substrate. The white scale bar indicates 600 nm. The image was taken after applying 10V in the outer square area (2 × 2 μm^2^) and then −10 V in the center square area (1 × 1 μm^2^). **(b)** Phase shift of the image obtained by averaging the data in the rectangle area surrounded by red lines in (a). The result shows that the ferroelectric polarization domains in the artificial superlattice BiFeO_3_/BiCrO_3_ thin film can be switched by the applied voltage.

The ferroelectric properties of the (1 1 1) orientated BiFeO_3_/BiCrO_3_ artificial superlattices were investigated by polarization domain switching using a scanning non-linear dielectric constant microscope. The measurements were performed at room temperature and an AC voltage of 0.5–2.0 V_p-p_ was applied between the back side of the substrate and the probe tip during the scans. Positively and negatively poled voltage patterns were observed in the scans of a 6-ML superlattice as shown in Figure 11. The result demonstrates that polarization reversal is indeed possible proving that the film is ferroelectric and that the ferroelectric polarization domains can be switched by the applied voltage. All the experimental results on ferromagnetic and ferroelectric properties suggest that the grown Bi_2_FeCrO_6_ artificial superlattice shows multiferroic property at room temperature.

## 3. Experimental Section

Bulk samples of Bi_2_NiMnO_6_ were prepared from a stoichiometric mixture of Bi_2_O_3_, NiO, and MnO_2_. The starting material was charged into a gold capsule, and treated at 6 GPa and 800 °C for 30 min in a cubic-anvil-type high-pressure apparatus. Then it was slowly cooled to room temperature for 4–50 h before releasing the pressure. The crystal structure of the material was analyzed by synchrotron powder X-ray diffraction with *λ* = 0.42098 Å. Diffraction patterns were collected with a large Debye-Scherrer camera installed at BL02B2 in SPring-8 [27]. The samples packed into glass capillaries were rotated during the measurements. High temperature diffraction data were also collected with temperature control equipment of the diffractometer to see the structural change by the ferroelectric transition. The measured diffraction patterns were refined by Rietveld method with the RIETAN-2000 program [28].

Bi_2_NiMnO_6_ thin films were synthesized by a pulsed laser deposition method with a KrF excimer laser (COHERENT COMPex-Pro 205 F, λ = 248 nm). A ceramic target was prepared by sintering from a stoichiometric mixture of Bi_2_O_3_, Mn_3_O_4_ and NiO. (0 0 1)-oriented SrTiO_3_ substrates were used. Substrate temperature and oxygen pressure during the deposition were 630 °C and 0.4 Torr, respectively. The films were deposited at a rate of 0.6 Å/sec to thicknesses of 500–1,000 Å. After the deposition, the films were cooled to 450 °C and annealed at that temperature for 1 h. The crystal structure of the obtained films was examined by X-ray diffraction. Thicknesses of the deposited films were also checked by Raue fringe in X-ray diffraction measurements. Synchrotron X-ray diffraction data was also collected in the reciprocal lattice space at room temperature in air by multi-axis diffractometers installed at BL13XU and BL46XU in SPring-8 [29]. An X-ray beam with a photon energy of 11.9 keV (wavelength; 0.104 nm) and a beam size of 0.1 × 0.1 mm^2^ was incident on the sample.

The Bi_2_FeCrO_6_ artificial superlattices were also fabricated at 500–600 °C by using pulsed laser deposition and alternately switching the BiFeO_3_ and the BiCrO_3_ ceramic targets. Two-dimensional growth was confirmed by monitoring the RHEED intensity *in situ* from the start of the deposition to the end. The deposition rate was about 0.1–0.3 Å/sec for both targets. SrTiO_3_ (1 1 1) substrates with atomically flat surfaces were used. A step-and-terrace structure with a step height of 1 unit cell was obtained by treating the substrate with a buffered hydrogen fluoride solution and then annealing it in flowing Ar gas.

The magnetic properties of the samples were measured with a superconducting quantum interference device (SQUID) magnetometer. The magnetic field was applied parallel to the film surface, and the contribution of the deposited thin film to the magnetization was estimated by subtracting the diamagnetic linear part due to the substrate.

The dielectric constant was measured with a conventional LCR meter with AC frequencies of 1 MHz. Ferroelectric properties of the Bi_2_NiMnO_6_ film samples were also measured for a *ϕ* 100 μm capacitor fabricated by conventional photolithography and Ar-milling techniques. Nb-0.05 wt% doped conductive SrTiO_3_ substrate was used as a bottom electrode and Pt-30 nm/Ti-30 nm/Au-50 nm was deposited as a top electrode. *P*(polarization)-*E*(electric field) hysteresis was recorded by measuring the polarization charge with a 5 kHz triangular waveform at various temperatures. The temperature dependence of the dielectric constant under a magnetic field was also measured with the LCR meter. The ferroelectric properties of the artificial superlattice were investigated using a scanning non-linear dielectric constant microscope [30]. The measurements were performed at 300 K, and an AC voltage of 0.5–2.0 V_p-p_ was applied between the back side of the substrate and the probe tip during the scans. The image was taken after applying 10V in the outer square area (2 × 2 μm^2^) and then −10 V in the center square area (1 × 1 μm^2^).

## 4. Conclusions

Multiferroic compounds with double-perovskite structures were synthesized. Bi_2_NiMnO_6_ was synthesized both in high-pressure synthesized bulk and epitaxially grown thin film forms. Bi_2_FeCrO_6_ was fabricated in a BiFeO_3_/BiCrO_3_ artificial superlattice with a 1/1 stacking period on a perovskite structure SrTiO_3_ (1 1 1) substrate. All the materials prepared showed both ferromagnetic and ferroelectric properties, that is, multiferroic properties at low temperature. The Bi^3+^ ion located at the A site in the perovskite structure caused ferroelectric structural distortion. B-site ordering of the Ni^2+^ and Mn^4+^ ions (Fe^3+^ and Cr^3+^ ions) in a rock-salt configuration led to ferromagnetism according to the Kanamori-Goodenough rule.

The high-pressure synthesized bulk of Bi_2_NiMnO_6_ showed a noncentrosymmetric *C*2 structure and a ferroelectric phase transition at 485 K. The sample also exhibited a ferromagnetic behavior below 140 K with the saturated magnetization of about 4.1 μ_B_/f.u., which was close to 5 μ_B_/f.u. expected from ferromagnetic ordering of Ni^2+^ (*S* = 1) and Mn^4+^ (*S* = 3/2).

The epitaxially grown Bi_2_NiMnO_6_ thin-film sample grown on a SrTiO_3_ substrate had a pseudo-tetragonal structure, and it also showed ferromagnetic properties. The clear *P*-*E* hysteresis loop was observed in the thin-film sample. Dielectric constant showed a very small anomaly at the magnetic transition temperature, suggesting an interplay between the ferromagnetic and ferroelectric interactions. The coupling between ferromagnetic and ferroelectric interactions seemed to exist in Bi_2_NiMnO_6_, but it was quite small.

(111)-oriented BiFeO_3_/BiCrO_3_ artificial superlattices up to 6-monolayer thick with one-by-one periodicity were fabricated by monitoring the layer-by-layer growth from the RHEED intensity oscillations. Both ferromagnetic behaviors and ferroelectric polarization switchings were confirmed, and it should be emphasized that the multiferroic property was observed at room temperature with the obtained artificial superlattice.
